# Digitally clinical and esthetic assessment on a modified connective tissue graft (epithelium island) technique

**DOI:** 10.3389/froh.2026.1889034

**Published:** 2026-10-23

**Authors:** Yue Liao, Han Hu, Huxiao Li, Huiwen Chen, Wentao Sun, Jiachen Dong, Zhongchen Song

**Affiliations:** 1 Department of Periodontology, Shanghai Ninth People’s Hospital, Shanghai Jiao Tong University School of Medicine, Shanghai, China; 2 College of Stomatology, Shanghai Jiao Tong University, Shanghai, China; 3 National Center for Stomatology, National Clinical Research Center for Oral Diseases, Shanghai Key Laboratory of Stomatology, Shanghai Research Institute of Stomatology, Shanghai, China

**Keywords:** CIEDE2000, connective tissue graft, epithelium island, intra-oral scanning, periodontal phenotype modification

## Abstract

**Background:**

The aim of this study was to evaluate the clinical efficacy and esthetic outcome of a modified connective tissue graft (the epithelium island, EI) technique for periodontal phenotype modification in patients undergoing orthodontic treatment via digital assessment of intra-oral scanning, cone beam computed tomography (CBCT), and the CIEDE2000 formula.

**Methods:**

Fifteen patients with RT-2 localized gingival recession and thin periodontal phenotypes were enrolled. Clinical parameters were assessed pre-surgery (T_0_) and 6 months post-surgery (T_3_). Digital evaluations using 3Shape TRIOS® intra-oral scanning and CBCT data were performed to measure keratinized tissue width (KTW), gingival thickness (GT), and root exposure area (REA). Gingival color difference (ΔE_00_) was objectively calculated by self-developed software (GAES®) based on the CIEDE2000 formula at T_0_, T_1_, and T_3_.

**Results:**

RD significantly decreased from 3.00 ± 0.76 mm (T_0_) to 0.90 ± 0.43 mm (T_3_) (*p* < 0.001), and RW reduced from 6.07 ± 0.80 mm to 3.73 ± 0.80 mm (*p* < 0.005). KTW increased by 2.85 ± 0.28 mm at T_3_ (*p* < 0.001). GT of affected teeth increased by 0.73 ± 0.11, 1.09 ± 0.34, and 1.03 ± 0.30 mm at 0, 1, and 3 mm apical to the gingival margin (T_3_) (*p* < 0.001). Meanwhile, the REA decreased from 5.85 ± 0.97 mm^2^ (T_0_) to 0.76 ± 0.42 mm^2^ (T_3_), a reduction of 5.10 ± 1.07 mm^2^ (*p* < 0.001). The ΔE_00_ values at T_3_ decreased by 0.865 ± 0.323 (free gingival margin) and 0.845 ± 0.372 (middle zone of keratinized gingiva) compared to T_0_ (*p* < 0.001).

**Conclusion:**

The EI technique effectively reduces gingival RD, RW, and REA, increases KTW and GT, and achieves favorable esthetic outcomes in patients with thin periodontal phenotypes during orthodontic treatment.

**Clinical relevance:**

This study verifies that the EI technique provides a predictable surgical option for periodontal phenotype modification, enabling simultaneous periodontal functional reconstruction and esthetic improvement for orthodontic patients with fragile gingival conditions.

## 1 Introduction

Orthodontic treatments are increasingly sought for esthetic and functional improvement, but the quantity and integrity of soft tissues are critical for the long-term stability of the periodontium during treatment (1). Patients with thin periodontal phenotypes are more likely to suffer from gingival recession (GR) than those with thick phenotypes, and most untreated gingival recessions in thin phenotypes continue to progress despite good oral hygiene (2, 3).

Notably, patients with thin periodontal phenotypes not only have thin gingiva but are also more prone to insufficient keratinized tissue (KT) (4, 5). Clinically, at least 2 mm of keratinized tissue width (KTW) is recommended to avoid periodontal problems, including the progression of gingival recession (6). For patients lacking sufficient gingiva or KT, periodontal phenotype modification (PPM) therapies (PhMTs) are a promising solution to increase the width and thickness of gingiva and KT. These therapies can reduce the risk of adverse outcomes associated with thin phenotypes (e.g., preventing the initiation and progression of clinical attachment loss) and contribute to optimal esthetic results by enhancing root coverage (7, 8). Thus, PhMTs are highly recommended for patients with severe GR and insufficient keratinized tissue to support orthodontic treatment (5, 9).

Coronally advanced flap (CAF) combined with subepithelial connective tissue graft (SCTG), a widely recognized PhMT, is considered the gold standard for treating GR. However, recent systematic reviews and meta-analyses indicate that the SCTG + CAF gold standard technique shows lower predictability in sites with inadequate baseline KTW or deep gingival recession defects, where complete root coverage (CRC) rates may decrease from the typical 70%–85% to around 40%–55% (10). For patients without sufficient KT, the traditional solution is a two-step technique first described by Bernimoulin et al. in 1975 (11): augmenting keratinized gingiva with a free gingival graft (FGG) in the apical region of the exposed root, followed by CAF to achieve root coverage approximately 2 months later. Zucchelli and De Sanctis (12) proposed an improved two-step technique, which includes two surgical procedures: either FGG combined with CAF for the affected tooth or FGG in adjacent teeth combined with laterally positioned flap (LPF) surgery.

The two-step technique might be a valid option in challenging cases where single-stage procedures are not feasible or predictable. However, the disadvantages of a time-consuming process and increased pain from the secondary operation are inevitable. Thus, addressing gingival recession in sites with insufficient KT while achieving favorable esthetic outcomes remains a clinical challenge.

To address these clinical issues, the epithelium island (EI) technique—a modified connective tissue graft technique—was designed to simultaneously increase the width of keratinized gingiva, achieve maximum root coverage, and enhance the gingival thickness (GT) of adjacent teeth. The soft tissue augmentation was assessed through clinical examination and digital evaluation based on intra-oral scanning (IOS) and cone beam computed tomography (CBCT) data, which were finely aligned using modules of Implant Studio (3Shape, Copenhagen, Denmark). The difference in gingival color among treated and adjacent teeth was evaluated using the self-developed software (Gingival Aesthetic Evaluation System®, GAES®). This approach aims to combine the keratinized tissue augmentation of FGG with the root coverage potential of SCTG while minimizing color and texture disparities.

Thus, this study evaluates the clinical efficacy and esthetic outcomes of the EI technique in modifying periodontal phenotypes during orthodontic treatment, with the goal of providing a viable solution for patients with insufficient KT and gingival recession.

## 2 Materials and methods

### 2.1 Patient selection

Fifteen patients were recruited from the Department of Periodontology, Shanghai Ninth People's Hospital, Shanghai Jiao Tong University School of Medicine, who were diagnosed with localized gingival recession. The inclusion criteria were as follows: (1) suffering from Cairo RT-2 localized gingival recession with insufficient keratinized tissue (KTW < 1 mm) or shallow vestibular depth; (2) no uncontrolled systemic diseases; (3) not being pregnant; (4) no smoking. The periodontal phenotype was evaluated using the periodontal probe transparency test with an UNC-15 (Hu-Friedy®, Chicago, IL, USA) metal probe (4).

The study was approved by the institutional board of Shanghai Ninth People's Hospital, Shanghai Jiao Tong University School of Medicine (Ethical consent NO. SH9H-2021-T414-1), and informed consent was signed by all the patients. The workflow diagram of the study is shown in Figure 1:

**Figure 1:**
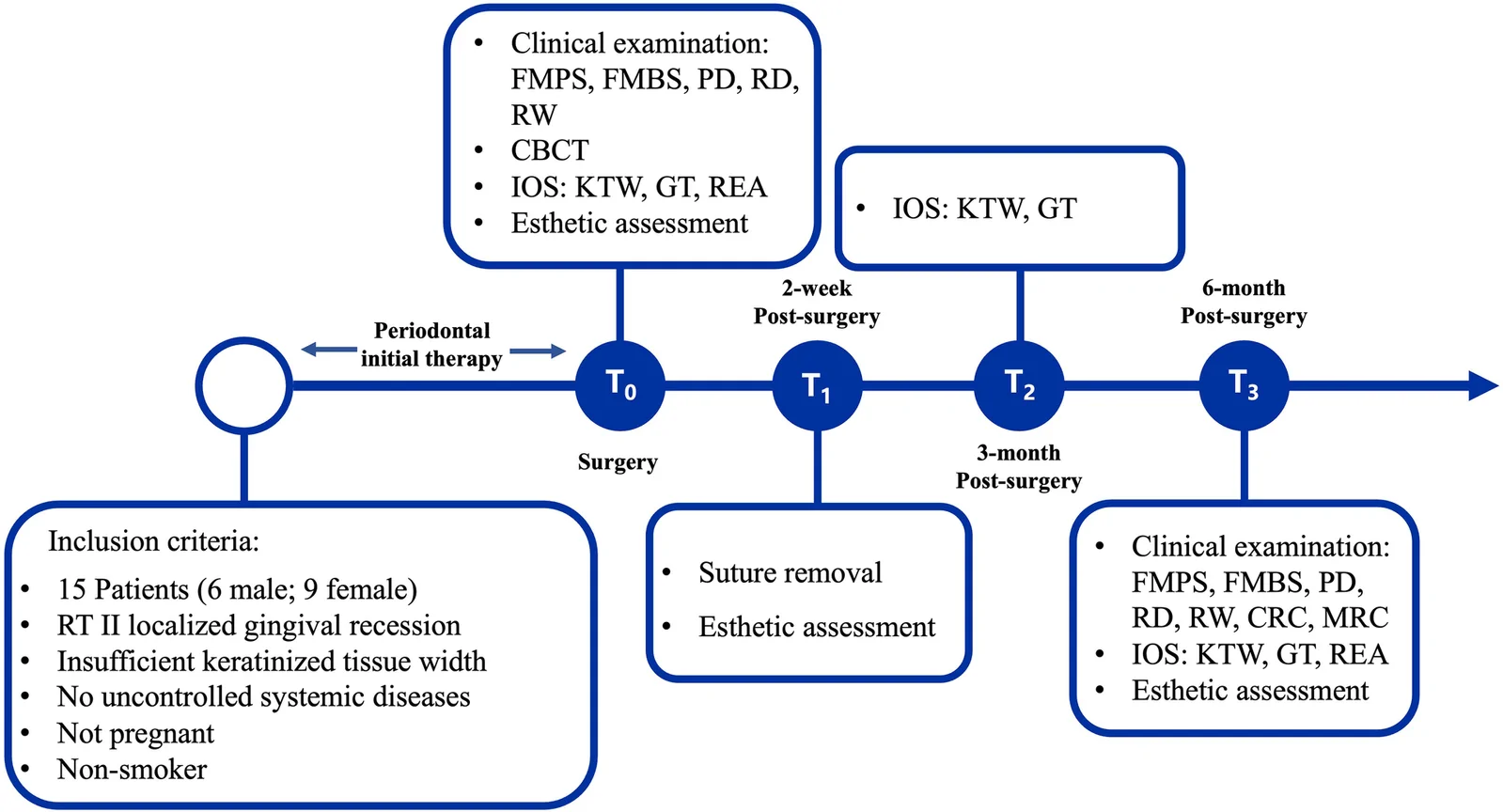
Workflow diagram of the study.

### 2.2 Clinical examination

A comprehensive periodontal assessment was performed before surgery. The full-mouth plaque score (FMPS) and full-mouth bleeding score (FMBS) were calculated after the initial therapy. Probing depth (PD, 6 sites/tooth), tooth movement (TM), recession depth (RD) of mid-buccal gingiva, recession width (RW), and FMBS and FMPS were measured pre-surgery (baseline, T_0_) and 6 months post-surgery (T_3_).

### 2.3 Digital evaluation by intra-oral scan and CBCT

Digital impressions of both the maxillary and mandibular arches were captured using the 3Shape TRIOS 4 intra-oral scanner (3Shape, Copenhagen, Denmark) pre-surgery (T_0_), at 3 months (T_2_), and at 6 months post-surgery (T_3_). The 3Shape TRIOS 4 scanner has an accuracy of ±10 μm. Scanning extended to the base of the vestibular sulcus and palatal mucosa to include all teeth and soft tissue.

The data from the IOS were exported and saved in STL format for analysis. CBCT data were acquired to evaluate the alveolar bone volume and to fuse with the IOS data. CBCT was performed with a voxel size of 0.125 mm, covering the entire surgical area. Registration and fusion of the IOS and CBCT data were performed using 3Shape TRIOS® software. First, IOS data (STL) and CBCT data were imported into the modules of Implant Studio, with the CBCT cropped and the intra-oral model trimmed to reduce interference. Then, at least three shared anatomical landmarks on dental hard tissues were manually marked for initial registration, followed by fine alignment using occlusal and labial feature areas. Finally, multi-planar rotation verified the fit between the roots and alveolar bone for secondary registration.

The pre- and post-surgery intra-oral scan data were superimposed to analyze KTW and soft tissue augmentation. GT was measured at 0, 1, and 3 mm apical to the gingival margin (GM) at the mid-buccal aspect of affected and adjacent teeth.

For the assessment of gingival recession area, the region of interest (ROI), representing the root exposure area (REA), was defined on the scanned models: the coronal boundary was set at the gingival margin, the apical boundary was determined by the most apical extension of the recession defect, and the mesial and distal boundaries were delineated according to the midline of the interdental papilla. After establishing a closed curved ROI along the anatomical boundaries, the surface area of the gingival recession was automatically calculated using 3Shape TRIOS® software (13, 14).

### 2.4 Surgical procedure

1) A horizontal incision was made at the mucogingival junction of the affected teeth, and a split-flap was prepared at the apical area of the incision (Figure 2A).
2) The coronal area of the horizontal incision was de-epithelialized (Figure 2B).
3) The EI was harvested from the palate (Figure 2C). The connective tissue graft portion (1 mm thick) was harvested using the L technique, while the other portion (2 mm thick) was harvested in the same manner as obtaining the epithelium-connective tissue graft. The length ratio of the epithelium-containing connective tissue part to the connective tissue part should be 1:1.5.
4) The epithelium portion of the graft was placed on the affected tooth and then sutured with horizontal cross mattress sutures. The connective tissue component was secured beneath the split-flap of adjacent teeth using interrupted sutures. The gingival flap of adjacent teeth can be coronally advanced with suspension sutures if gingival recession occurs (Figure 2D).

**Figure 2:**
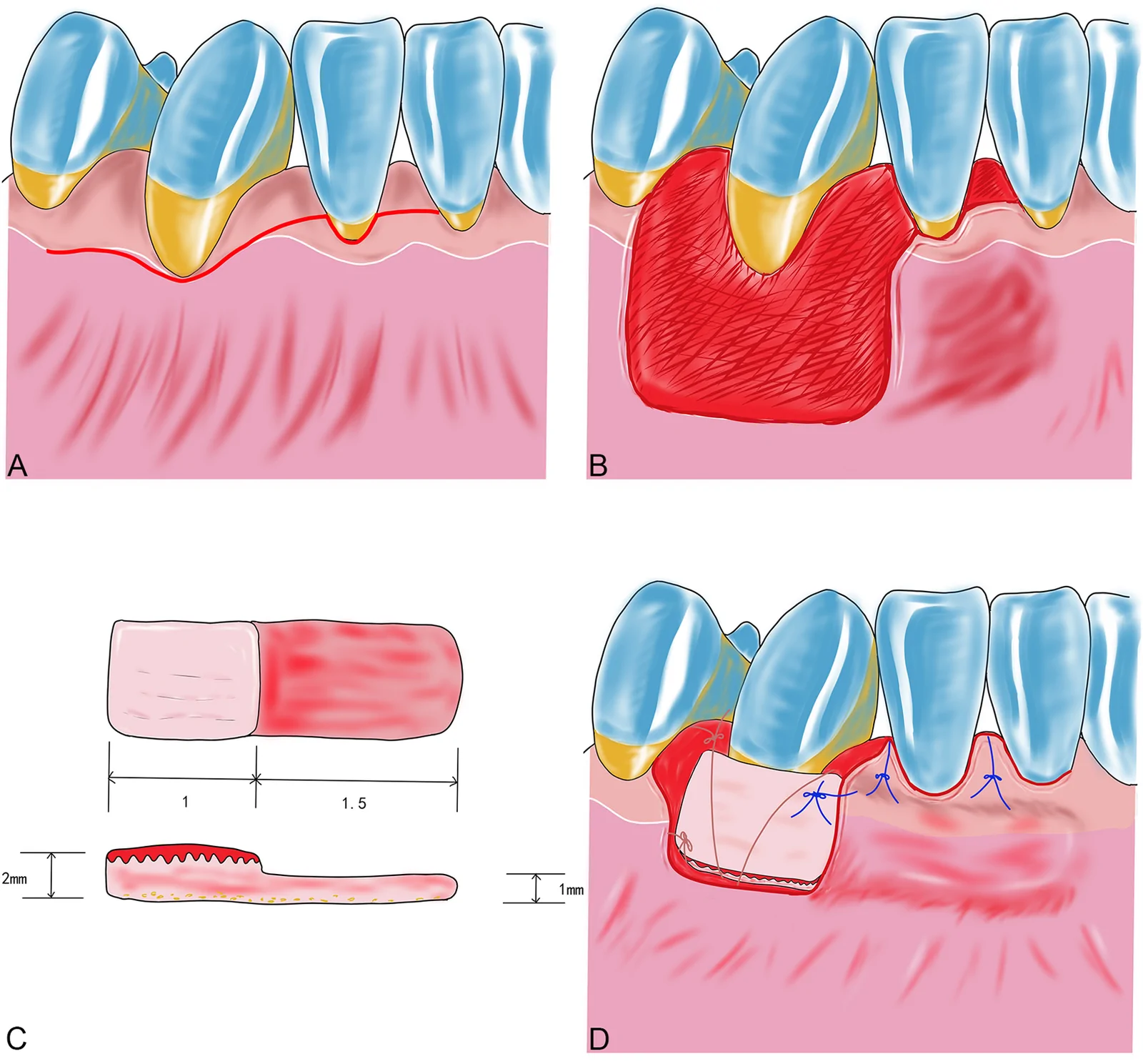
Schematic diagram of the surgical procedure. **(A)** Design of the horizontal incision, **(B)** de-epithelization of the coronal region of the incision, **(C)** harvest of EI graft, and **(D)** suturing of the graft and gingival flap.

All patients underwent initial periodontal therapy to achieve a full-mouth plaque score and full-mouth bleeding score of ≤20% (Figures 3A,B). The split-flap (Figures 3C,D) was created at adjacent teeth after assessing maximum root coverage (MRC). Then, the coronal area of the horizontal incision was de-epithelialized, and the epithelium island graft was harvested (Figures 3E,F). The graft and gingival flap were sutured as previously described (Figure 3G). The sutures were removed 2 weeks later (Figure 3H), with follow-up visits at 3 and 6 months postoperatively (Figure 3I).

**Figure 3:**
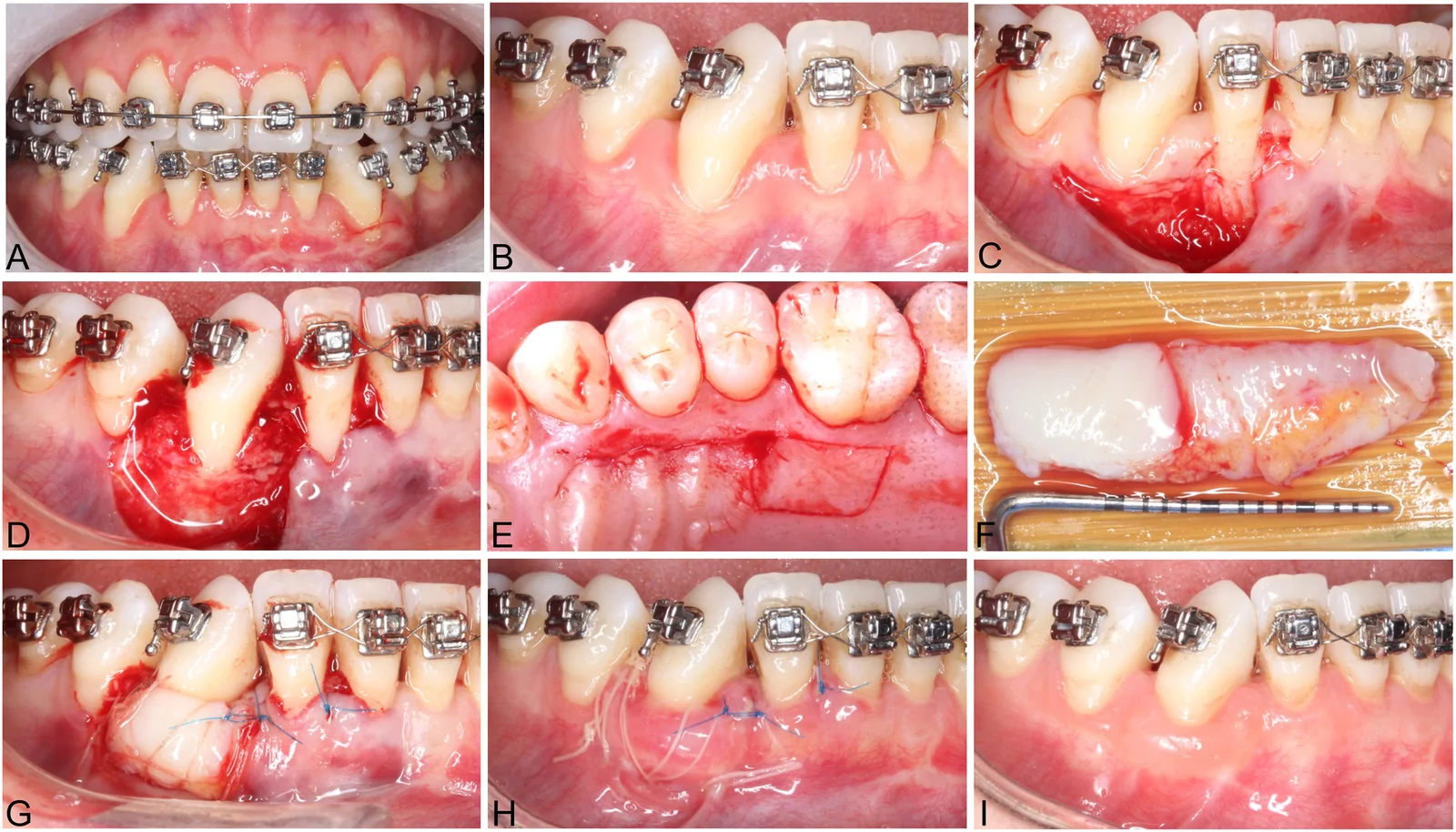
Surgical procedure of connective tissue with partly epithelium graft (EI) at #42-43. **(A)** Intra-oral frontal view pre-surgery, **(B)** intra-oral view of #43 showing gingival recession and insufficient keratinized tissue, **(C)** horizontal incision of #43, **(D)** de-epithelialized coronal area of the horizontal incision, **(E,F)** harvesting connective tissue with partly epithelium graft (EI), **(G)** postoperative view after suturing, **(H)** suture removal 2 weeks post-surgery, and **(I)** intra-oral view of #42-43 at 6 months post-surgery.

### 2.5 Esthetic assessment

Gingival color evaluations were performed pre-surgery (T_0_), 2 weeks post-surgery (T_1_), and 6 months post-surgery (T_3_) by one periodontist who did not participate in the surgical treatment and was blinded to the initial esthetic status of the patients. Color coordinates were measured using zenithal fluorescent daylight lamps that simulate D65 illumination at 6500 K, following ISO/TR 28642.

The color differences of the gingiva were compared between affected teeth and adjacent teeth at the free gingival margin (FGM) and the middle zone of keratinized gingiva (MZ) on the labial side (15) (Figure 4). The gingival color difference (ΔE_00_) values were calculated by the self-developed software (GAES®) via the CIEDE2000 formula (16).

**Figure 4:**
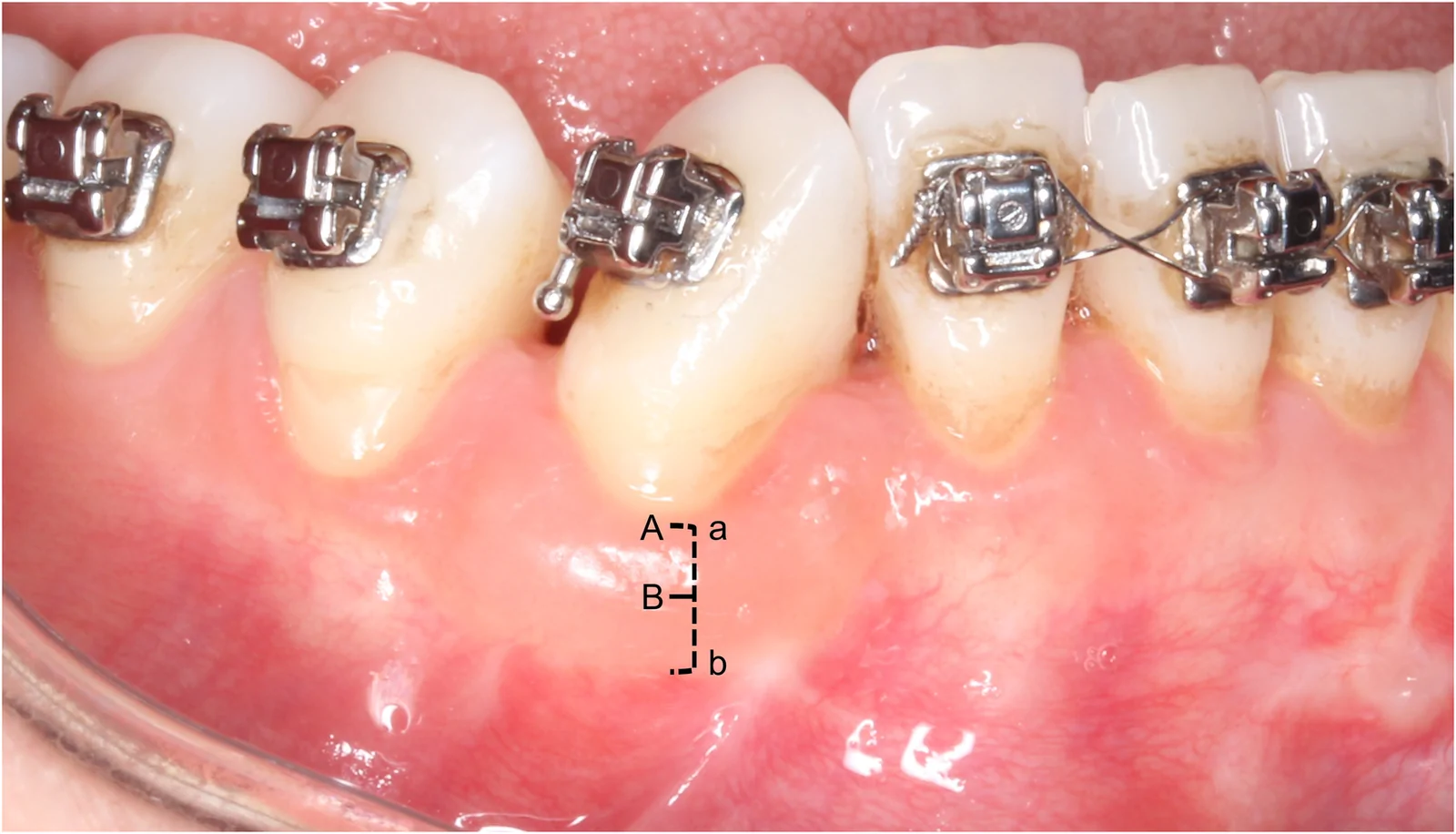
Measurement points of gingiva. A: FGM. B: MZ. a–b: keratinized tissue width.

### 2.6 Statistical analysis

Quantitative variables of clinical parameters and esthetic assessments were expressed as averages and standard deviations. Comparisons of the values of L*, a*, and b* were conducted using independent *t*-tests. One-way ANOVA was used to compare changes in KTW, GT, and gingival color differences. We constructed a 95% confidence interval for Hedges’ g using the non-central t-distribution. A *p* < 0.05 was considered statistically significant. All statistical analyses were performed using SPSS software (version 29.0).

## 3 Results

### 3.1 Patient characteristics

This study enrolled 15 patients (9 females, 6 males) with a mean age of 27.60 ± 6.16 years, with one affected tooth per patient (*n* = 15 teeth total). All patients were diagnosed with RT-2 localized gingival recession, accompanied by insufficient KTW (<1 mm) at the recession defect and a thin periodontal phenotype. Regarding tooth distribution, 4 (26.67%) affected teeth were located in the maxilla and 11 (73.33%) in the mandible (Table 1).

**Table 1:** Patient characteristics at baseline

Variables	Mean±SD or number (percentage)
Age (years)	27.60±6.16
Gender	
Female	9 (60%)
Male	6 (40%)
Jaw type (n=15)	
Maxillary	4 (26.67%)
Mandibular	11 (73.33%)

### 3.2 Clinical periodontal parameters

All periodontal parameters were measured by a specialized and calibrated periodontist. PD remained consistent pre- and post-surgery, with no statistically significant difference (T_0_: 2.00 ± 0.64 mm; T_3_: 2.07 ± 0.54 mm; *p* = 0.594). Both FMPS and FMBS were less than 20% at baseline (T_0_) and 6 months post-surgery (T_3_) (FMPS: 16.25% ± 2.11% vs. 16.37% ± 1.63%, *p* = 0.863; FMBS: 16.07% ± 1.97% vs. 16.38% ± 1.86%, *p* = 0.658), indicating that patients maintained good oral hygiene throughout the study period.

Gingival RD and RW decreased significantly after surgery. RD decreased from 3.00 ± 0.76 mm at baseline to 0.90 ± 0.43 mm at T_3_ (*p* < 0.001). In addition, RW reduced from 6.07 ± 0.80 mm (T_0_) to 3.73 ± 0.80 mm (T_3_) (*p* < 0.001). Detailed clinical parameters are presented in Table 2.

**Table 2:** Clinical periodontal parameters (mean ± SD) pre-surgery (T_0_) and 6-month follow-up (T_3_)

Parameter	T_0_	T_3_	*p*-value
PD (mm)	2.00 ± 0.64	2.07 ± 0.54	0.594
RD (mm)	3.00 ± 0.76	0.90 ± 0.43	<0.001
RW (mm)	6.07 ± 0.80	3.73 ± 0.80	<0.001
FMPS (%)	16.25 ± 2.11	16.37 ± 1.63	0.863
FMBS (%)	16.07 ± 1.97	16.38 ± 1.86	0.658

### 3.3 Digital analysis of gingival thickness

GT was quantitatively analyzed using 3Shape TRIOS software, with pre- and post-surgery IOS data superimposed to visualize morphological changes (Figure 5). Gingival tissue with a GT increase of >0.3 mm is displayed in yellow, and >0.8 mm in red (Figure 5D).

**Figure 5:**
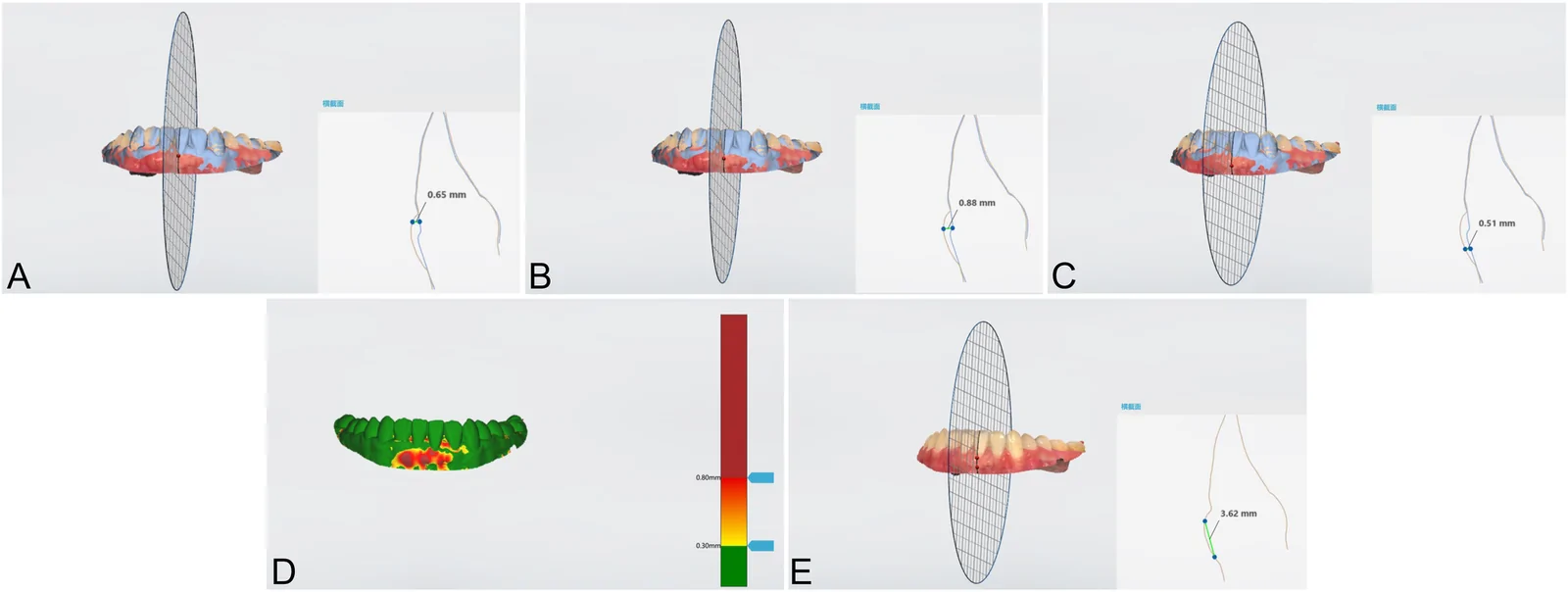
Digital analysis of GT and KTW by intra-oral scan. **(A)** Change in GT at the gingival margin. **(B)** Change in GT 1 mm apical to the gingival margin. **(C)** Change in GT 3 mm apical to gingival margin. **(D)** Pre- and post-treatment IOS overlay, illustrating morphological changes in the surgery area. **(E)** Keratinized tissue width measured by the IOS image.

For affected teeth, GT at the GM increased from 0.59 ± 0.16 mm (T_0_) to 1.28 ± 0.27 mm at 3 months post-surgery (T_2_) and remained stable at 1.31 ± 0.20 mm at T_3_. At 1 mm apical to GM, GT increased from 0.60 ± 0.18 mm (T_0_) to 1.70 ± 0.33 mm (T_2_) and 1.69 ± 0.36 mm (T_3_). At 3 mm apical to GM, GT increased from 0.59 ± 0.16 mm (T_0_) to 1.62 ± 0.38 mm (T_2_) and 1.63 ± 0.39 mm (T_3_) (Figures 6A–C).

**Figure 6:**
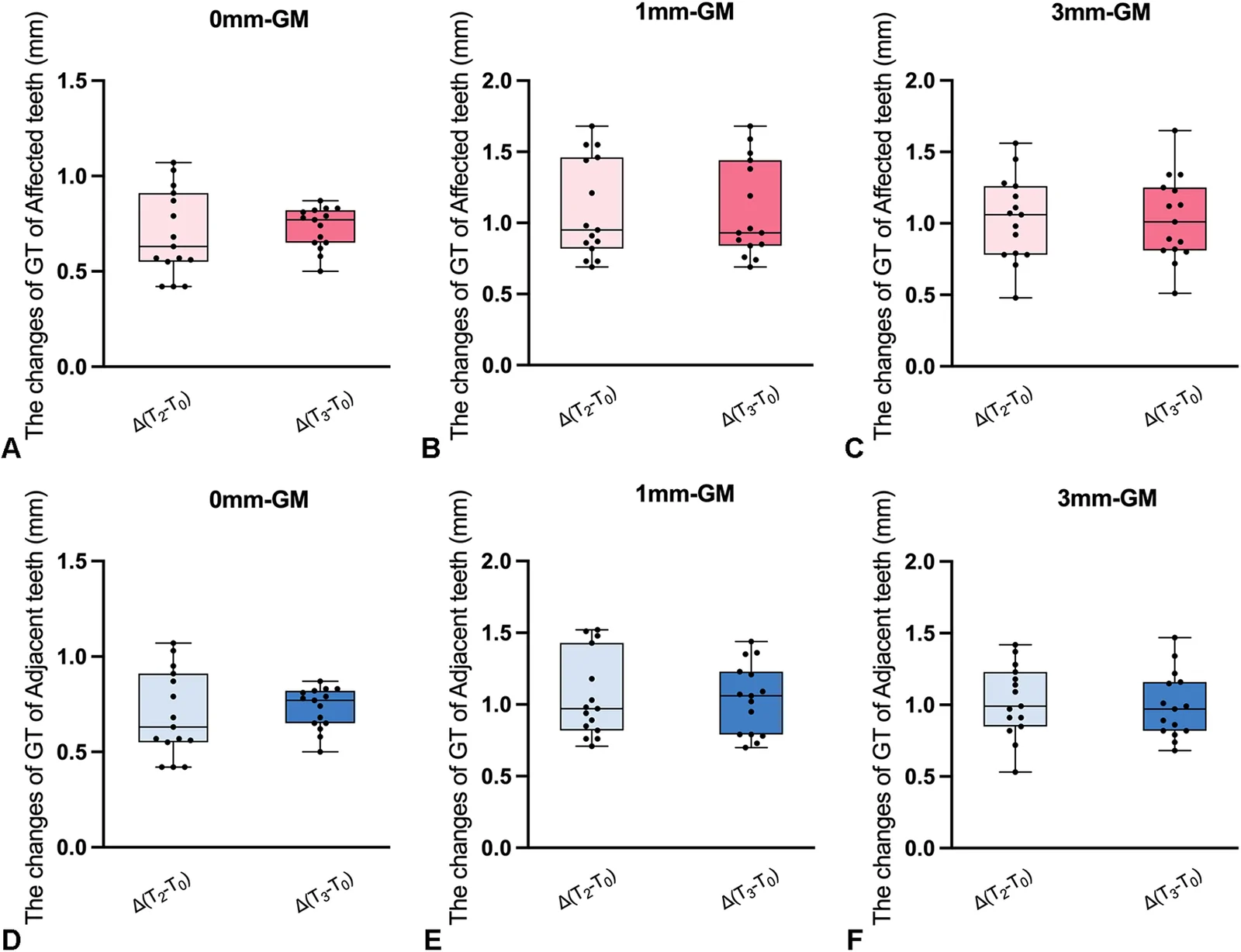
Changes in GT (mean ± SD) 0, 1, and 3 mm apical to gingival margin pre-surgery (T_0_), 3-month (T_2_), and 6-month follow-up (T_3_). The changes in GT for affected teeth **(A–C)** and adjacent teeth **(D–F)**.

For adjacent teeth, similar increases in GT were observed at all three measurement points. At GM, GT increased from 0.58 ± 0.17 mm (T_0_) to 1.32 ± 0.19 mm (T_2_) and 1.34 ± 0.18 mm (T_3_). At 1 mm apical to GM, GT increased from 0.59 ± 0.18 mm (T_0_) to 1.64 ± 0.32 mm (T_2_) and 1.63 ± 0.30 mm (T_3_). At 3 mm apical to GM, GT increased from 0.62 ± 0.14 mm (T_0_) to 1.65 ± 0.30 mm (T_2_) and 1.62 ± 0.28 mm (T_3_) (Figures 6D–F). Notably, the maximum absolute increase in GT (ΔGT) was observed at 1 mm apical to GM in both affected and adjacent teeth, with all increments showing statistical significance (*p* < 0.001).

### 3.4 Digital analysis of keratinized tissue width

KTW was measured using IOS data (Figure 5E). At baseline (T_0_), the mean KTW of affected teeth was 0.60 ± 0.12 mm. After surgery, KTW increased continuously, reaching 3.23 ± 0.25 mm at T_2_ and further to 3.45 ± 0.31 mm at T_3_ (*p* < 0.001). The absolute change in KTW (ΔKTW) was 2.63 ± 0.23 mm (T_2_ − T_0_) and 2.85 ± 0.28 mm (T_3_ − T_0_), respectively, with statistically significant differences compared to baseline (*p* < 0.05) (Figure 7A). Meanwhile, the values of REA decreased from 5.85 ± 0.97 mm^2^ (T_0_) to 0.76 ± 0.42 mm^2^ (T_3_), a reduction of 5.10 ± 1.07 mm^2^ (*p* < 0.001) (Figure 7B).

**Figure 7:**
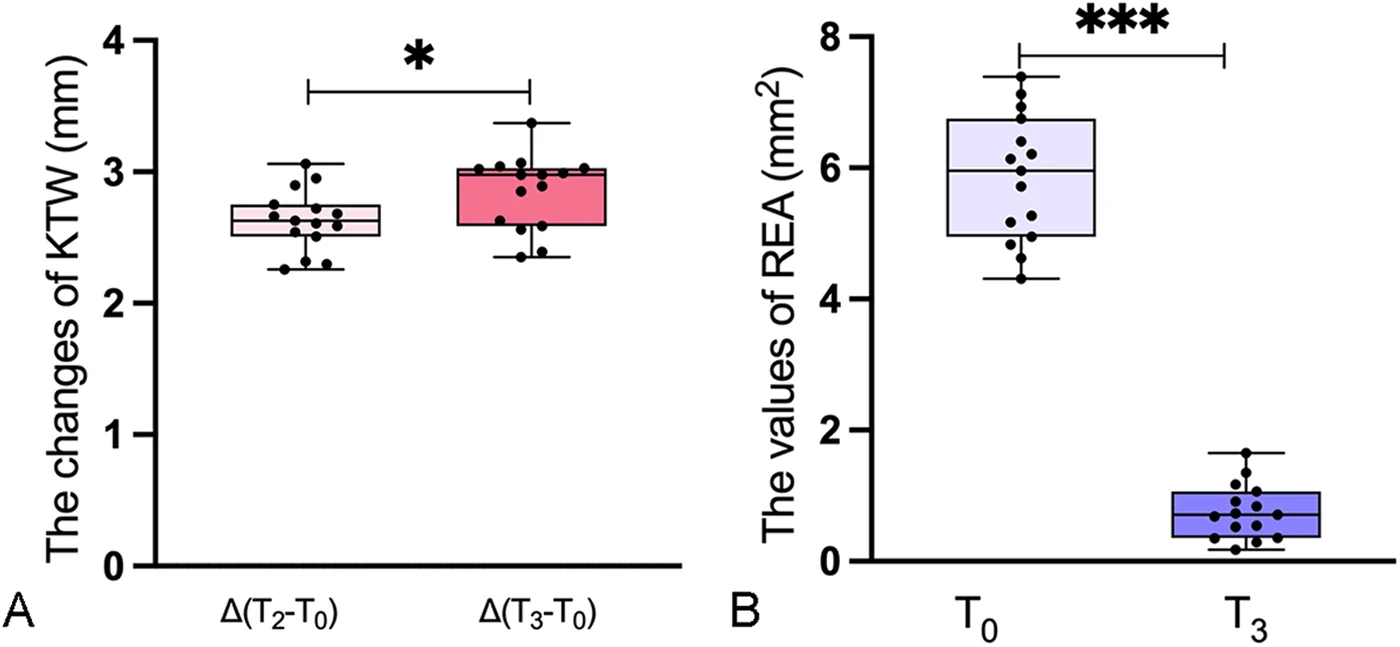
Changes in KTW **(A)** and the values of REA **(B)** for affected teeth (mean ± SD). ***p < 0.001; *p < 0.05.

### 3.5 Esthetic outcome

The gingival color difference (ΔE_00_) values were evaluated by GAES® software based on the CIEDE2000 formula (Figure 8). In the CIELAB color space, L* (lightness), a* (red-green axis), and b* (yellow-blue axis) values of affected teeth showed no significant differences compared with adjacent teeth at T_0_, 2 weeks post-surgery (T_1_), and T_3_ (*p* > 0.05), indicating consistent color harmony in these dimensions throughout the study period.

**Figure 8:**
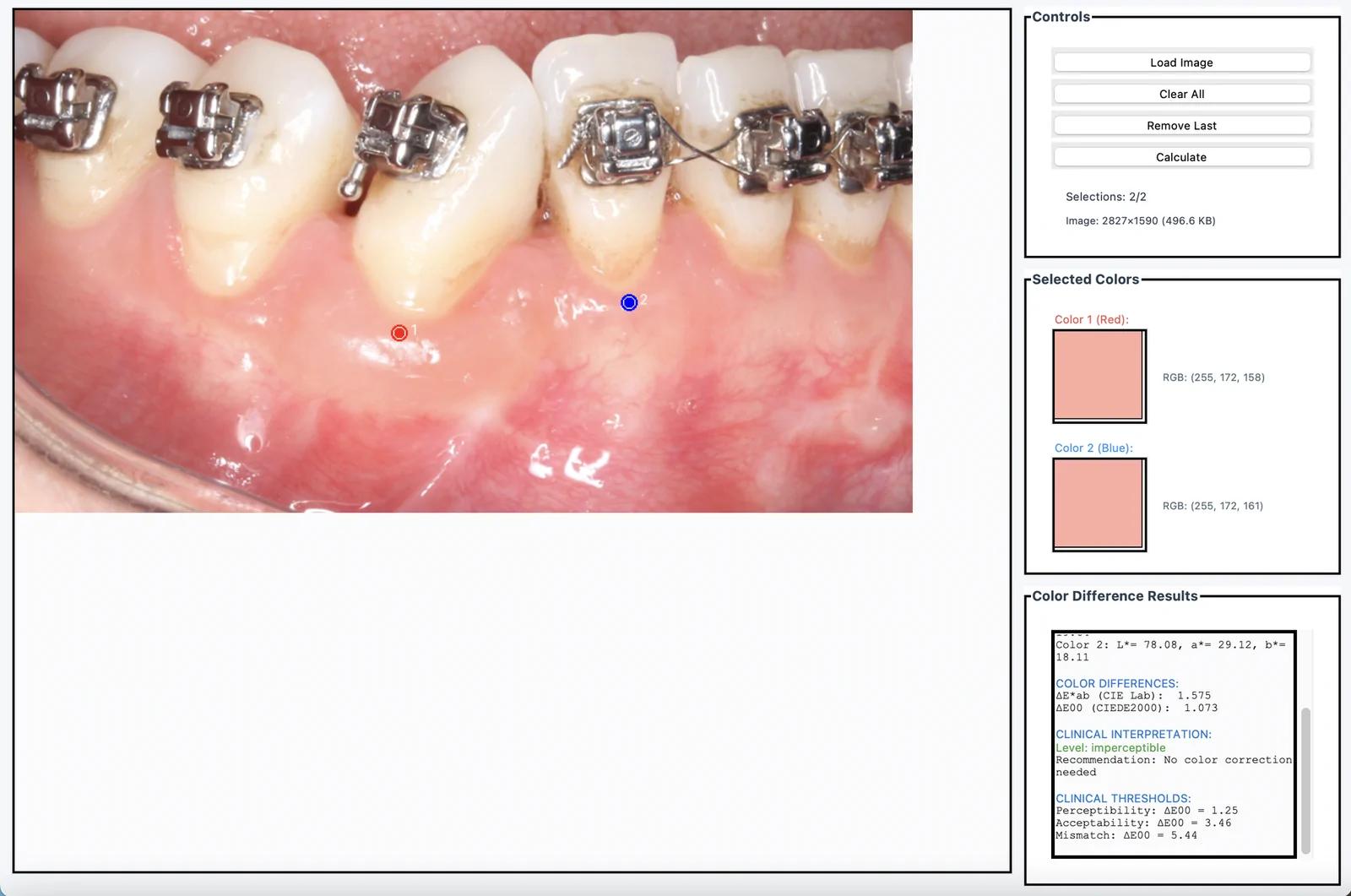
Interface of GAES® software for ΔE_00_ value calculation.

ΔE_00_ values at T_1_ (suture removal) were 2.12 ± 0.34 (FGM) and 2.09 ± 0.28 (MZ), similar to baseline values (T_0_: FGM = 2.09 ± 0.28; MZ = 2.18 ± 0.36). At T_3_, ΔE_00_ values significantly decreased to 1.22 ± 0.16 (FGM) and 1.34 ± 0.21 (MZ) (*p* < 0.001), representing a reduction of 0.865 ± 0.323 (FGM) and 0.845 ± 0.372 (MZ) compared with T_0_. All ΔE_00_ values at T_0_, T_1_, and T_3_ were ≤2.7, meeting the criterion for an “acceptable color match” (Tables 3, 4).

**Table 3:** The color difference of the gingiva (ΔE_00_) at Baseline (T_0_), 2-week (T_1_), and 6-month follow-up (T_3_).

CIELAB color space	Site	T_0_		T_1_		T_3_	
		FGM	MZ	FGM	MZ	FGM	MZ
L*	Affected tooth	75.53±4.60	74.93±7.80	70.40±5.79	71.13±8.81	71.87±6.13	76.60±8.81
	Adjacent tooth	77.07±4.03	74.93±7.54	69.80±5.94	70.47±9.55	72.40±6.33	76.20±7.93
	*p*	0.339	1	0.782	0.844	0.816	0.897
a*	Affected tooth	28.80±7.97	27.47±7.21	33.93±11.66	51.67±7.43	29.73±5.26	28.33±7.37
	Adjacent tooth	30.93±8.73	28.80±7.70	33.87±10.13	51.20±8.39	29.60±5.01	28.20±7.39
	*p*	0.490	0.628	0.987	0.873	0.133	0.961
b*	Affected tooth	15.47±6.69	15.80±6.87	19.00±8.08	19.80±7.66	17.00±6.38	14.60±7.95
	Adjacent tooth	15.87±6.81	16.00±6.41	18.80±8.30	20.47±8.00	16.67±5.51	15.13±8.19
	*p*	0.872	0.935	0.947	0.817	0.879	0.858
ΔE_00_	Value	2.09±0.28	2.18±0.36	2.12±0.34	2.09±0.28	1.22±0.16	1.34±0.21
	*p*			＜0.001^a^	＜0.001^a^	＜0.001^b^	＜0.001^b^

^a^The comparison of ΔE_00_ between T_1_–T_0_.

^b^The comparison of ΔE_00_ between T_3_–T_0_.

**Table 4:** The change in ΔE_00_ values between T_1_ and T_0_, T_3_ and T_0_, and T_3_ and T_1_.

ΔE_00_	Mean		SD		95% confidence interval			
	FGM	MZ	FGM	MZ	FGM		MZ	
ΔE_00_ (T_1_–T_0_)	0.033	−0.095	0.530	0.382	−0.260	0.327	−0.307	0.116
ΔE_00_ (T_3_–T_0_)	−0.865	−0.845	0.323	0.372	−1.044	−0.686	−1.051	−0.640
ΔE_00_ (T_3_–T_1_)	−0.899	−0.564	0.378	0.488	−1.108	−0.689	−0.710	−0.417

## Discussion

Patients with a thin periodontal phenotype face a higher risk of gingival recession during and after orthodontic treatment, making periodontal phenotype optimization a critical clinical need (1, 2). Autogenous soft tissue grafts, including FGG and SCTG, are the preferred options for PPM (7). FGG effectively increases KTW but yields unpredictable root coverage outcomes (17) and often causes conspicuous white scarring, leading to significant esthetic concerns (18). The CAF combined with SCTG—known as the bilaminar technique—is regarded as the “gold standard” for achieving CRC and mean root coverage (MRC) with favorable esthetics (10). However, existing studies on this technique have primarily included patients with baseline KTW >1.3 mm, indicating that insufficient preoperative KTW may limit its effectiveness (5, 9).

For regions with gingival recession and insufficient keratinized tissue, two-step techniques have emerged as alternative options (11, 12). Nevertheless, color mismatch with adjacent gingiva remains a major drawback, which may worsen over long-term healing and compromise the final esthetic outcome. Clinicians have attempted to address the limitations of FGG through modified techniques such as the partly epithelialized FGG (PE-FGG) (19) and gingival unit graft (20), aiming to simultaneously increase KTW and achieve root coverage in a single procedure. However, these modified approaches still have inherent limitations, including inadequate KTW augmentation or unclear GT increments. Furthermore, comprehensive digital evaluations of clinical parameters and quantitative esthetic outcomes have not been fully reported for these techniques.

In this study, the EI technique—a novel modified connective tissue graft—was developed. In this technique, the epithelial portion of the graft is designed to increase keratinized tissue and achieve root coverage, while the connective tissue portion enhances GT. Unlike conventional FGG, the connective tissue component of the EI graft is covered by a gingival flap, ensuring superior blood supply and promoting graft survival. The epithelial portion, supported by the underlying connective tissue, resembles an “island,” hence the name “epithelium island (EI) technique.” The EI technique demonstrates potential advantages over conventional alternatives in the specific indication of RT-2 gingival recession with baseline KTW <1 mm in orthodontic patients. Specifically, it achieves comparable KTW augmentation to FGG and comparable GT increment to CAF + SCTG in a single surgical procedure, while avoiding the color mismatch and scar formation associated with FGG.

All patients in this study had a thin periodontal phenotype with baseline KTW of 0.60 ± 0.12 mm. After treatment with the EI technique, KTW increased by 2.85 ± 0.28 mm over 6 months, reaching 3.45 ± 0.31 mm (*p* < 0.001). In addition, the values of RD, RW, and REA decreased, accompanied by a significant increase in GT. These findings confirm that the EI technique can simultaneously achieve reliable root coverage and KTW augmentation, addressing the unmet clinical needs of patients with insufficient keratinized tissue.

In the present study, the precise and quantitative analysis of clinical parameters (GT, KTW) pre- and post-surgery was based on data acquired from digital IOS and CBCT. The integration of IOS-derived three-dimensional soft tissue morphology and CBCT-based hard tissue information enables accurate, non-invasive, and reproducible evaluation of gingival contour, dimension, and volumetric alterations, effectively overcoming the subjectivity, invasiveness, and low consistency inherent in traditional manual probing and qualitative assessment methods. As validated in recent investigations, the fusion of IOS and CBCT data significantly improves the accuracy and reliability of periodontal soft tissue evaluation, providing a robust digital foundation for surgical outcome assessment (21, 22). With the continuous advancement of deep learning and multimodal medical image processing, artificial intelligence (AI) has emerged as a promising tool for automated tissue segmentation, registration, and measurement. Therefore, further research will be conducted to establish an AI-driven automatic detection system dedicated to assessing pre- and postoperative periodontal parameters, which is expected to enhance the efficiency, objectivity, and intelligence of surgical evaluation and support refined clinical decision-making and long-term prognostic monitoring in periodontal therapy.

In terms of esthetic assessment, self-developed software (GAES®) was used to calculate the gingival color difference (ΔE_00_) between affected and adjacent teeth. As per the current ISO/CIE (IOS-J03) standard (23), CIEDE2000 provides more accurate evaluations of human gingival color difference thresholds compared to other formulas. Previous studies have defined ΔE_00_ ≤ 1.1 as an “excellent match,” ΔE_00_ ≤2.7 as an “acceptable match,” and ΔE_00_ >5.4 as an “unacceptable mismatch” (24). In the present study, ΔE_00_ values at 2 weeks post-surgery (T_1_) were close to baseline (T_0_), while at 6 months post-surgery (T_3_), ΔE_00_ values decreased significantly, indicating that the gingival color of affected teeth became more consistent with adjacent teeth over time. Specifically, ΔE_00_ values at T_0_ (FGM: 2.09 ± 0.28; MZ: 2.18 ± 0.36), T_1_ (FGM: 2.12 ± 0.34; MZ: 2.09 ± 0.28), and T_3_ (FGM: 1.22 ± 0.16; MZ: 1.34 ± 0.21) all fell within the “acceptable match” range (≤2.7), confirming the favorable esthetic outcomes of the EI technique.

This study has several limitations that should be acknowledged. First, the sample size was small (15 patients), and the follow-up duration was limited to 6 months. Second, the lack of a control group (e.g., comparison with traditional techniques such as FGG or CAF + SCTG) prevents direct evaluation of the EI technique's superiority. Future studies should enroll larger cohorts, extend the follow-up period to 1–2 years to assess long-term stability, and adopt a randomized controlled design to compare the EI technique with conventional approaches. In addition, exploring the application of the EI technique in more complex anatomical scenarios (e.g., multiple adjacent recessions, severe vestibular shallowness) could further expand its clinical utility.

In conclusion, the EI technique offers a novel, single-stage solution for modifying periodontal phenotypes in patients with gingival recession and insufficient keratinized tissue during orthodontic treatment. By integrating the advantages of traditional grafting techniques and addressing their key limitations, it achieves significant improvements in gingival recession, KTW, and GT, along with favorable esthetic outcomes. The digital evaluation methods and objective color analysis (GAES® software) used in this study enhance the reliability and clinical relevance of the findings, supporting the potential of the EI technique to become a valuable tool in periodontal clinical practice.

## Data Availability

The raw data supporting the conclusions of this article will be made available by the authors, without undue reservation.
